# Ingestion of titanium dioxide as an excipient in medicines and the risk of cancer: a nationwide study within the French National health data system

**DOI:** 10.1007/s10654-025-01263-4

**Published:** 2025-07-02

**Authors:** Manon Cairat, Gianluca Severi, Inge Huybrechts, Agnès Fournier

**Affiliations:** 1 Université Paris-Saclay, UVSQ, Inserm, Gustave Roussy, CESP, 94805 Villejuif, France; 2 Department of Statistics, Computer Science and Applications “G. Parenti”, University of Florence, Firenze, Italy; 3 Nutrition and Metabolism Branch, International Agency for Research on Cancer, Lyon, France

**Keywords:** Titanium dioxide, Cancer, Pharmaceutical excipient, Medico-administrative database, Pharmacoepidemiology

## Abstract

Concerns about the safety of titanium dioxide (TiO_2_), including potential carcinogenicity, have prompted its ban in foods in the European Union, while remaining allowed as pharmaceutical excipient. We aimed to evaluate whether ingesting increasing quantities of TiO_2_ through medicines is associated with higher cancer risk. Data were derived from the French National Health Data System, a nationwide medico-administrative database. A case-control study was nested within two cohorts: users of metformin (all doses) and users of 200 mg acebutolol, both available in TiO_2_-containing and TiO_2_-free formulations. During 2013–2021, 293,101 cancer cases were identified and matched to 2,930,633 controls. TiO_2_ exposure through metformin and acebutolol consumption was calculated based on drug claims from 2006 up to five years before the index date. Conditional logistic regression models estimated linear associations between TiO_2_ exposure and cancer risk. RRs of overall cancer per 1000 TiO_2_-containing tablets and per 10,000 mg of TiO_2_ increments were both 1.00 (95% CI: 0.99–1.01). Analyses by cancer site also yielded RRs very close to 1.00 or slightly different but not statistically significant, except for breast (RR per 10,000 mg: 1.03, 95% CI:1.00-1.07) and lymphoid/hematopoietic (RR per 1000 tablets: 0.97, 95% CI: 0.95-1.00) cancers, which however lost significance after Bonferroni correction. There was a suggestion of non-linear positive association for central nervous system cancers. This first epidemiological study on TiO_2_ ingestion and cancer found no meaningful linear association between increasing TiO_2_ exposure through medicines and overall or site-specific cancer risk. Non-linear associations cannot be excluded.

**Supplementary Information:**

The online version contains supplementary material available at 10.1007/s10654-025-01263-4.

## Introduction

Food-grade titanium dioxide (TiO_2_) is widely used as pharmaceutical excipient for its ability to enhance whiteness and opacity, improve contrast with colorants, and protect photosensitive active ingredients from light degradation [1]. In France, 57% of oral pharmaceutical specialties on the market in 2020 contained TiO_2_ [2]. In foods, TiO_2_ is used as a whitening and brightening agent, for example in candies, chewing gums, baked goods, skimmed milk, white sauces and icings [3]. The comparison between estimated daily TiO_2_ intake from drug consumption in France and realistic estimates of dietary intake in European populations suggests that TiO_2_ exposure from pharmaceuticals is lower than dietary exposure in adults aged 20–59, but may reach comparable levels in older individuals [2].

In May 2021, the European Food Safety Authority (EFSA) concluded that, based on the available evidence and associated uncertainties, TiO_2_ could no longer be considered safe for use in foods. That conclusion was supported by findings from experimental studies suggesting immunotoxicity, inflammation and neurotoxicity, and potential genotoxicity [3]. Furthermore, in one study, TiO_2_ ingestion promoted preneoplastic lesions expansion in the colon of rats [4]. TiO_2_ could be carcinogenic through mechanisms such as oxidative stress, inflammation, and genotoxicity [5]. Food-grade TiO_2_ contains nanoparticles, characterized by their small size and large surface area, in varying proportions (approximately 10 to 50%) [6]. These nanoparticles may contribute to the potential health effects of TiO_2_, given their ability to cross biological barriers and induce oxidative stress, DNA damage, apoptosis and inflammatory responses [7, 8].

In January 2022, based on EFSA’s assessment—particularly the unresolved concern regarding genotoxicity and the many uncertainties—the European Commission decided to ban the use of TiO_2_ as a food additive [9, 10]. Since EFSA did not identify an immediate health risk and to allow for a smooth transition, foods containing TiO_2_ were permitted to be placed on the market until August 2022 and to be sold until their date of minimum durability or ‘use by’ date.

Although banned in foods, TiO_2_ remains provisionally authorized in medicines in the European Union [9, 10]. That decision was based on the European Medicines Agency’s scientific analysis regarding the technical purpose of the use of TiO_2_ in medicinal products and the feasibility and timelines for its replacement [1], and aimed to avoid shortages, as the replacement of TiO_2_ would require investigation and testing of suitable alternatives. A review clause in the Regulation stipulates that the European Commission must assess whether to maintain or remove TiO_2_ as an excipient in pharmaceuticals within three years of the Regulation’s entry into force —that is, in 2025—based on an updated analysis by the European Medicines Agency.

However, there is a lack of epidemiological studies examining the long-term health effects of TiO_2_ ingestion in humans, including on cancer risk. We thus performed an epidemiological study to investigate whether the ingestion of increasing quantities of TiO_2_ through medicines is associated with higher cancer risk, on the assumption that any increase in TiO_2_ exposure would cause a monotonic variation in cancer risk.

## Materials and methods

### Data sources

This study used the French National Health Data System (SNDS, *Système National des Données de Santé*), which contains, for over 99% of the French population, individual-level medico-administrative data on outpatient services reimbursed by the National Health Insurance as well as hospital discharge diagnoses and long-term diseases (LTD) registrations (e.g., for cancer) [11, 12]. Drug claim data include dispensing dates for reimbursed medications together with their French presentation identification codes. The SNDS also provides demographic information such as sex, dates of birth and death, and place of residence. Hospital discharge diagnoses include “main” (e.g. myocardial infarction, chemotherapy) and “related” (e.g. breast cancer) diagnoses, as well as “associated” diagnoses, i.e. patient’s comorbidities involving a higher burden of care. All data can be linked using a pseudonymized identifier. The SNDS integrates data from several insurance schemes, the “General scheme” for salaried workers covering 75% of the population with data recorded since 2006. Smaller schemes have been progressively incorporated.

We also used data from the French Theriaque database (https://www.theriaque.org) as of 2021, which provides, for all pharmaceutical products ever marketed in France, detailed information including presentation identification codes, the list of excipients—including TiO_2_—, pharmaceutical form, strength, Anatomical Therapeutic Chemical (ATC) codes, and package dispensing unit numbers. Data on exact TiO_2_ quantities, obtained from the French medicines safety agency (ANSM) as of 2021, were available for 42% of TiO_2_-containing pharmaceutical specialties [2].

### Selection of drugs

To minimize confounding related to participants’ characteristics (see Discussion), we restricted our analyses to users of selected drugs. Drugs were characterized by their active molecule, strength, and pharmaceutical form, and were selected based on these criteria:

1. Availability in both TiO_2_-containing and TiO_2_-free formulations.
2. At least 300,000 boxes of both TiO_2_-containing and TiO_2_-free formulations reimbursed annually between 2006 and 2016. This threshold was determined a posteriori to ensure sufficient precision in estimating the TiO_2_-cancer associations (see Results) while limiting the number of selected drugs.
3. No excipient was exclusively present in either TiO_2_-containing or TiO_2_-free formulations, allowing us to disentangle the effects of TiO_2_ from effects of other excipients.

Applying these criteria, we initially selected: metformin (ATC code A10BA02) tablets at doses of 500, 850, and 1000 mg, tablets combining levonorgestrel 150 µg and ethinylestradiol 30 µg (G03AA07), tablets of acebutolol (C07AB04) 200 mg, ibuprofen (M01AE01) 200 mg, and doxycycline (J01AA02) 100 mg (see also Supplementary Methods and Table S1 in Online Resource). We excluded ibuprofen and doxycycline due to their predominant short-term use, limiting relevance for dose-response analyses. Additionally, the levonorgestrel-ethinylestradiol combination was excluded because the presence of TiO_2_ and carnauba wax were strongly correlated, precluding isolation of TiO_2_’s effects.

We combined all doses of metformin tablets to create a unique cohort of metformin users. Ultimately, the selected drugs were:

1. Metformin tablets (all doses).
2. Acebutolol 200 mg tablets.

### Cohorts of selected drug users

For each selected drug (metformin and 200 mg acebutolol), we assembled a cohort of users by including individuals affiliated with the “General scheme” with at least one claim for the selected drug in the SNDS over 2006–2021. Follow-up for cancer incidence began on January 1, 2013, or the date of the first identified delivery of the selected drug, whichever was later. The earliest follow-up start date was January 1, 2013, because individuals in the “General scheme” who died before 2013 may not have had their unique identifier integrated into the SNDS [11]. Follow-up ended at the earliest of death, first recorded cancer diagnosis, or December 31, 2021.

We excluded individuals with a cancer diagnosis or death before follow-up started, and those whose first claim for the selected drug occurred after follow-up ended. The acebutolol 200 mg and metformin cohorts comprised 587,164 (Figure S1 in Online Resource) and 3,155,100 (Figure S2 in Online Resource) individuals, respectively.

### Identification of cancer cases

Cancer occurrence was identified by:

- a hospital discharge diagnosis with an ICD-10 code for cancer (codes starting with ‘C’ or ‘D0’), considering “main”, “related”, or “associated” diagnoses, or
- an LTD registration with a cancer ICD-10 code.

The retained date of cancer diagnosis was the earliest of either an LTD registration for cancer or a hospital discharge with a cancer code.

Cancer cases were classified by site using ICD-10 main categories. Subcategories were also used (e.g., ovarian cancer within the “Malignant neoplasms of female genital organs” main category), provided they accounted for at least 15% of cases within the main category (Table S2 in Online Resource).

### Selection of cases and controls

We implemented a nested case-control design within each cohort of selected drug users.

For each cancer case diagnosed during follow-up, up to ten controls were randomly selected using incidence density sampling. Controls were required to be cancer-free on the case’s diagnosis date (index date) and to meet the following matching criteria: date of first delivery of the selected drug identified in the SNDS (± 6 months), year of birth, and sex. Cases could serve as controls prior to their diagnosis. Thereby, the odds ratio is an unbiased estimate of the incidence rate ratio (RR) that would be obtained in a cohort study conducted within the source population [13].

In analyses where TiO_2_ exposure was characterized by cumulative dose rather than the cumulative number of tablets, cases with an unknown cumulative dose at diagnosis (i.e., if a drug box with an unknown TiO_2_ amount had been purchased at any time before diagnosis) were excluded. The same matching procedure as above was applied, with the additional requirement that controls had a known cumulative dose at the case’s diagnosis date.

The corresponding flow charts are shown as Figures S1 and S2 (Online Resource).

### Exposure to titanium dioxide

Deliveries of the selected drug (metformin or acebutolol 200 mg, depending on the cohort of origin of the individual) were identified in the SNDS using French presentation identification codes. Cumulative TiO_2_ exposure was calculated up to five years before the index date (5-year lag), to avoid considering exposures unlikely to influence cancer risk, given cancer development latency periods [14]. Individuals whose first delivery of the selected drug occurred less than 5 years before the index date were excluded. Cumulative exposure to TiO_2_ was quantified as:

- The number of TiO_2_-containing tablets of the selected drug reimbursed from drug claims data availability (2006) to five years before the index date.
- The cumulative TiO_2_ dose (in mg) in the selected drug tablets reimbursed during the same period.

### Covariates

Adjusted analyses included the French geographical social deprivation index [15] and geographical region (Table 1, and Table S3 in Online resource) at the time of the first selected drug claim. We also calculated the cumulative number of selected drug tablets containing the individual constituents listed in Table 1, from 2006 to five years before the index date.

**Table 1:** Main characteristics of cancer cases and matched controls

	Case-control study with exposure measured as cumulative number of TiO_2_-containing tablets		Case-control study with exposure measured as cumulative dose of TiO_2_ in milligrams	
	Cases *n* = 293,101	Controls *n* = 2,930,633	Cases *n* = 218,611	Controls *n* = 2,185,643
*Cohort of origin (matching factor)*				
Metformin	231,167 (78.9%)	2,311,494 (78.9%)	180,345 (82.5%)	1,803,217 (82.5%)
Acebutolol 200 mg	61,934 (21.1%)	619,139 (21.1%)	38,266 (17.5%)	382,426 (17.5%)
Time since first use of the selected drug, mean (SD), years	9.6 (2.8)	9.6 (2.8)	9.4 (2.8)	9.4 (2.8)
*Sociodemographic parameters*				
Age at index date, mean (SD), years (matching factor)	73.4 (10.4)	73.4 (10.3)	73.2 (10.3)	73.2 (10.3)
Sex (matching factor)				
Male	178,587 (60.9%)	1,785,640 (60.9%)	133,774 (61.2%)	1,337,429 (61.2%)
Female	114,514 (39.1%)	1,144,993 (39.1%)	84,837 (38.8%)	848,214 (38.8%)
French social deprivation index, quintiles				
1 (less deprived)	38,898 (13.6%)	411,228 (14.6%)	27,580 (13.0%)	290,180 (13.8%)
2	59,621 (20.9%)	593,608 (21.0%)	43,431 (20.4%)	433,065 (20.6%)
3	67,904 (23.8%)	655,522 (23.2%)	50,899 (23.9%)	491,967 (23.4%)
4	85,423 (29.9%)	834,628 (29.6%)	64,013 (30.1%)	624,227 (29.7%)
5 (most deprived)	33,427 (11.7%)	325,367 (11.5%)	26,700 (12.6%)	260,329 (12.4%)
Missing	7828	110,280	5988	85,875
*Constituents contained in the selected drug*, *mean (SD)*, *cumulative number of tablets (lagged by 5 years)*				
Metformin 1000 mg, polyvinylpyrrolidone, and HPM	954 (1872)	926 (1838)	1130 (2020)	1097 (1987)
Metformin 850 mg and HPM	682 (1427)	678 (1415)	557 (1266)	554 (1259)
Metformin 500 mg and HPM	328 (901)	327 (902)	277 (793)	276 (794)
Acebutolol 200 mg, colloidal anhydrous silica, polyvinylpyrrolidone, magnesium stearate, polyethylene glycol, and HPM	257 (788)	253 (776)	176 (637)	171 (624)
Microcrystalline cellulose	66 (328)	64 (321)	7 (91)	7 (87)
Hydroxypropyl cellulose	260 (686)	255 (673)	217 (635)	212 (623)
Colloidal anhydrous silica	333 (856)	328 (842)	178 (634)	175 (623)
Talc	531 (1003)	517 (981)	485 (975)	472 (953)
Stearic acid	343 (836)	332 (814)	316 (832)	305 (810)
Magnesium stearate	1882 (2290)	1848 (2264)	1940 (2314)	1904 (2290)
Polyvinylpyrrolidone	946 (1590)	941 (1579)	831 (1475)	827 (1467)
Propylene glycol	52 (312)	52 (306)	48 (302)	48 (296)
Polyethylene glycol	1231 (1851)	1206 (1826)	1199 (1866)	1173 (1840)
Ethyl copolacmethacrylacryl	52 (327)	53 (328)	49 (324)	50 (325)
Croscarmellose sodium, iron oxide yellow, and polyacrylate	35 (225)	34 (218)	30 (214)	28 (206)
Sodium carboxymethyl starch and corn starch	55 (316)	55 (310)	48 (302)	48 (296)
Aromatic mixture^a^	4 (86)	5 (93)	5 (97)	5 (98)
Simethicone	22 (175)	21 (167)	18 (162)	17 (154)
Wheat starch	67 (339)	68 (341)	61 (335)	61 (336)
*Exposure to TiO*_*2*_ *through the selected drug (lagged by 5 years)*				
Cumulative amount of TiO_2_, mean (SD), milligrams^b^	4365 (9946)	4275 (9800)	4365 (9946)	4270 (9783)
Cumulative number of tablets containing TiO_2_, mean (SD)	1090 (1553)	1066 (1528)	917 (1481)	891 (1452)

*HPM* hydroxypropylmethylcellulose; SD, standard deviation

^a^ Sulfur dioxide, gum arabic, butylated hydroxyanisole, geranial, limonene, corn, myrcene, maltodextrin, neral, alpha-pinene, beta-pinene, sodium saccharin, sodium benzoate, and gamma-terpinene

^b^ The cumulative amount of TiO_2_ was missing for 74,448 (25.4%) cases and 751,205 (25.6%) controls in the case-control study with exposure measured as cumulative number of TiO_2_-containing tablets, and was complete for all individuals in the case-control study with exposure measured as cumulative dose of TiO_2_ in milligrams

## Statistical analyses

Conditional logistic regression models estimated RRs and 95% confidence intervals (CIs) for the association between cumulative TiO_2_ exposure and cancer risk. The two case-control databases (metformin and 200 mg acebutolol) were combined for a single case-control analysis conditioned on the matched pairs used in the individual case-control datasets.

We estimated RRs for incremental increases in TiO_2_ exposure (per 1000 tablets or per 10,000 mg) by including the exposure metric as a continuous variable in the models. We also obtained a graphical representation of the dose-response relationship between TiO_2_ exposure and the risk of cancer, by coding cumulative TiO_2_ exposure using restricted cubic splines with knots at the 5th, 50th, 75th and 95th percentiles [16].

All models were adjusted for the covariates mentioned earlier. The cumulative number of selected drug tablets containing each constituent listed in Table 1 was modeled with restricted cubic splines (knots at the 5th, 50th and 95th percentiles among controls exposed to that constituent).

Supplementary analyses included lagging exposure by one year instead of five and performing separate analyses of the metformin and acebutolol case-control datasets. In a sensitivity analysis, incident cancer cases were identified using LTD registrations as well as “main” and “related” diagnoses at hospital discharges, but not “associated” diagnoses.

All statistical tests were two-sided. Analyses were carried out with SAS Enterprise Guide software, version 8.3.

## Results

Table 1 and Table S3 (Online Resource) show the numbers and characteristics of cases and matched controls in the case-control study with cumulative number of TiO_2_-containing tablets and in the one with cumulative dose of TiO_2_ as exposure. The mean time between the first claim for the selected drug and the index date was approximately 9.5 years. Of the 3 billion TiO_2_-containing tablets of metformin and acebutolol 200 mg controls were exposed to, the information on the TiO_2_ content was missing for 20%, was ≤ 1 mg for 29%, > 1 to ≤ 5 mg for 16%, > 5 to ≤ 10 mg for 34%, and > 10 mg for 0.1% (4.7 mg on average among TiO_2_-containing tablets with a known TiO_2_ quantity). The corresponding figures were 20%, 30%, 16%, 34% and 0.1% (average 4.6 mg) among cases.

**Fig. 1:**
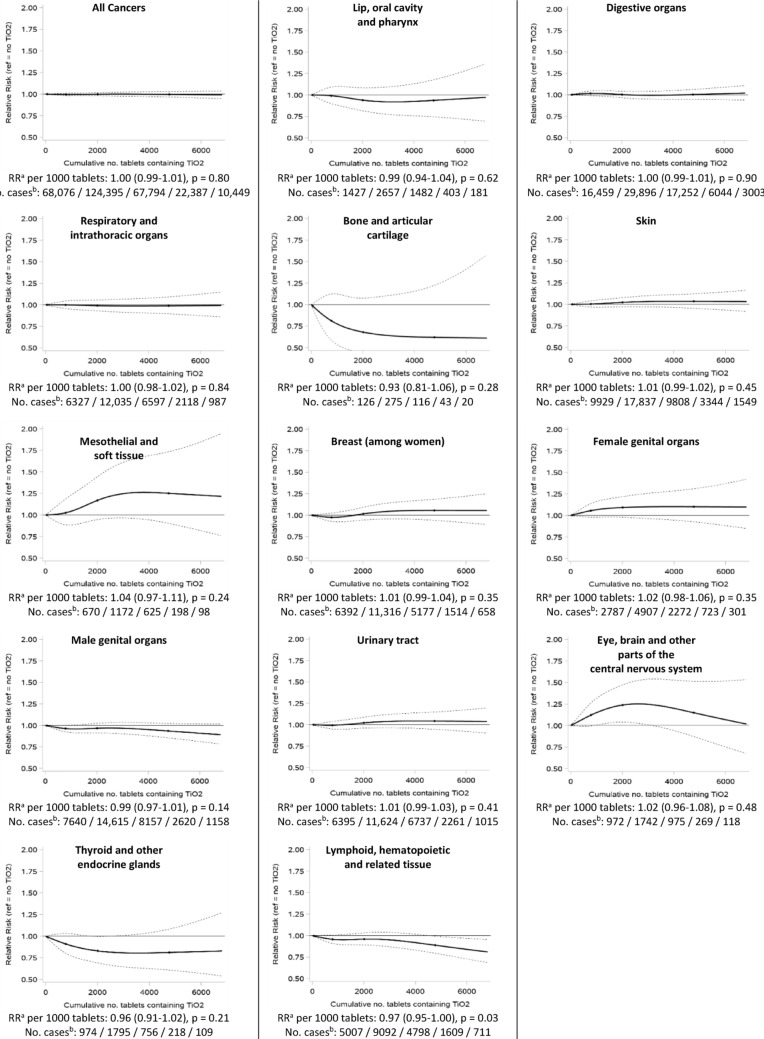
Associations between the cumulative number of TiO_2_-containing tablets ingested and the risk of cancer, overall and by main ICD-10 categories of cancer localizations. Exposure to TiO_2_ lagged by 5 years. *CI*, confidence interval; RR, incidence rate ratio. ^a^ Adjusted for age, sex, cohort of origin (metformin / acebutolol) and time since first use of the selected drug (metformin or acebutolol, depending on the cohort of origin) (matching factors), and region, social deprivation index, cumulative numbers of tablets containing the constituents listed in Table 1. ^b^ Number of cases among individuals exposed to 0 / 1-1000 / 1001–3000 / 3001–5000 / 5001 + tablets containing TiO_2_

**Fig. 2:**
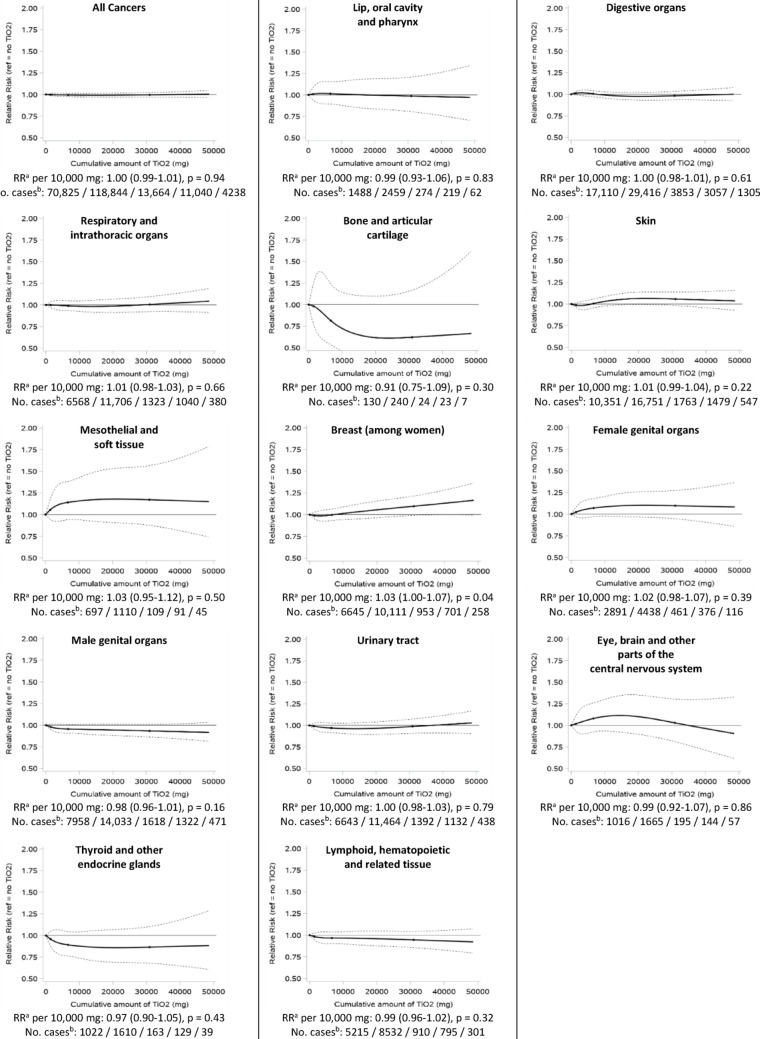
Associations between the cumulative quantity of TiO_2_ ingested (milligrams) and the risk of cancer, overall and by main ICD-10 categories of cancer localizations. Exposure to TiO_2_ lagged by 5 years. * CI* confidence interval; RR, incidence rate ratio. ^a^ Adjusted for age, sex, cohort of origin (metformin / acebutolol) and time since first use of the selected drug (metformin or acebutolol, depending on the cohort of origin) (matching factors), and region, social deprivation index, cumulative numbers of tablets containing the constituents listed in Table 1. ^b^ Number of cases among individuals exposed to 0 / 1–10,000 / 10,001–20,000 / 20,001–40,000 / 40,001 + mg of TiO_2_

Figures 1 (cumulative number of TiO_2_-containing tablets as the exposure metric) and 2 (TiO_2_ cumulative dose as the exposure metric) show the graphical representation of the dose-response relationships between TiO_2_ exposure and cancer risk, overall and by main ICD-10 categories, as well as the RRs associated with incremental increases in TiO_2_ exposure, assessing linear associations. The RRs of overall cancer per 1000 TiO_2_-containing tablets and per 10,000 mg of TiO_2_ increments were both 1.00 (95% CI: 0.99–1.01). RRs were also not statistically different from 1 at the *p* = 0.05 level among men and among women, and in age at index date strata (≤ 50 / 50–65 / 65–80 / 80 + years old) (data not shown). Analyses according to main cancer localizations also showed RRs that were very close to 1.00 (between 0.98 and 1.02 for lip, oral cavity and pharynx; digestive organs; respiratory and intrathoracic organs; skin; female genital organs; male genital organs; urinary tract; eye, brain and other parts of the central nervous system) or slightly further from the null but statistically non-significant at the *p* = 0.05 level (bone and articular cartilage; mesothelial and soft tissue; thyroid and other endocrine glands) (Figs. 1 and 2), indicating no meaningful linear associations. Only breast (RR per 1000 tablet increment: 1.01, 95% CI: 0.99–1.04; per 10,000 mg: 1.03, 95% CI:1.00-1.07) and lymphoid, hematopoietic and related tissue (RR per 1000 tablet increment: 0.97, 95% CI: 0.95-1.00; per 10,000 mg: 0.99, 95% CI: 0.96–1.02) cancers showed RRs that were statistically significant at the *p* = 0.05 level, although close to 1. There was a non-linear association between increasing cumulative number of TiO_2_-containing tablets and eye, brain and other parts of the central nervous system cancer risk (p_linearity_ = 0.03), with a higher risk for up to approximately 3000 tablets (Fig. 1).

TiO_2_ exposure showed no meaningful linear associations with the risk of cancer by localization subcategories (Figure S3 in Online Resource), except for ovarian cancer for which there was some suggestion of linearly increased risk with increasing exposure amount (RR per 1000 tablet increment: 1.05, 95% CI: 0.97–1.14; per 10,000 mg: 1.10, 95% CI:1.00-1.22). Non-linear associations were apparent for pancreatic cancer and melanoma (p_linearity_ = 0.02 for TiO_2_ cumulative dose as the exposure metric).

When accounting for multiple testing based on the number of tested cancer localizations using Bonferroni, all the above-mentioned associations lost statistical significance. The corrected significance level was indeed 0.004 when considering the 13 main cancer localizations, and 0.003 when also considering cancer sub-localizations.

Applying a 1-year instead of a 5-year lag for exposure assessment only marginally modified the estimates for linear associations (Table S4 in Online Resource). Analyses performed separately in the case-control studies nested in the cohort of metformin users and in the cohort of 200 mg acebutolol users showed concordant RRs per increment of 1000 TiO_2_-containing tablets, but the comparison of RRs per increment of 10,000 mg of TiO_2_ was hampered by the low precision of estimates among acebutolol users (Table S5 in Online Resource). Models that did not adjust for the exposure to constituents (active ingredients and excipients) other than TiO_2_ yielded RRs of cancer associated with increasing TiO_2_ exposure that were in most cases slightly higher than those obtained from fully adjusted models (Table S6 in Online Resource). A sensitivity analysis in which “associated” diagnoses at hospital discharges were not considered for the identification of incident cancer cases led to the same conclusions as our main analysis regarding linear associations (Table S7 in Online Resource).

## Discussion

This is the first epidemiological study assessing the association between TiO_2_ ingestion and cancer risk. Our findings revealed no meaningful linear association between increments in TiO_2_ exposure from medications and overall or site-specific cancer risk. The RRs of overall cancer per 1000 TiO_2_-containing tablets and per 10,000 mg of TiO_2_ increments were both 1.00 (95% CI: 0.99–1.01). Considering the median TiO_2_ amount in TiO_2_-containing tablets in France is 1.4 mg [2], ingesting 10,000 mg of TiO_2_ corresponds to about 7000 tablets. However, there was a suggestion of non-linear positive association for eye/brain/other parts of the central nervous system cancer.

TiO_2_ particles can cross the intestinal barrier and accumulate in various organs [17–19]. While systemic absorption after ingestion is low (< 0.5%), there is persistence and tissue accumulation of internalized particles, with 200–450 days half-lives [3]. TiO_2_ nanoparticles can also cross the blood-brain barrier [20]. Two experimental studies in animals raised concerns about the carcinogenicity of food-grade TiO_2_ ingestion [4, 21]. Bettini et al. observed that oral exposure to food-grade TiO_2_ in rats at a dose of 10 mg/kg body weight (BW)/day promoted colon micro-inflammation and initiated preneoplastic lesions while also fostering the growth of aberrant crypt foci [4]. Urrutia-Ortega et al. observed that intragastric administration of food-grade TiO_2_ at 5 mg/kg BW/day in mice was unable to induce tumor formation but could potentiate intestinal tumor [21]. However, observations on aberrant crypt foci were not replicated in subsequent studies administering food-grade TiO_2_ at doses up to 1000 mg/kg BW/day [3]. For comparison, we recently estimated that in France, the median amount of TiO_2_ per one tablet or capsule of TiO_2_-containing drug was 1.4 mg [2]. Based on national drug reimbursement data from 2012 to 2020, we further estimated that the average daily exposure to TiO_2_ from medicines was 1.71 mg per person — 1.24 mg among people aged 20–59 years old and 4.00 mg among people 60 years or older. These values correspond to approximately 0.02 mg/kg BW/day in adults and 0.05 mg/kg BW/day in the elderly. Our epidemiological study found no increased risk of colon cancer with increasing ingestion of TiO_2_ through medications.

The effects of TiO_2_ on health may depend on its characteristics, including crystalline structure and particle size. According to the European Pharmacopoeia, TiO_2_ used in medicinal products corresponds to food-grade TiO_2_ (European designation: E171), which is highly pure but not subject to specific EU limits regarding particle size. A 2019 EFSA report based on data from manufacturers showed that E171, typically composed of anatase or rutile forms, contains varying proportions of nanoparticles [6]. In five commercial anatase products (anatase being the form primarily used in pharmaceuticals [22]), the median particle size ranged from 104 to 166 nm, with 11.4–45.6% of particles < 100 nm by number. These data highlight the variability and uncertainty surrounding the particle size distribution of TiO₂ in medicines. This is supported by analyses from two French consumer associations, which reported nanoparticle contents in TiO₂ ranging from 11 to 44% across several commonly used drug products, with notable discrepancies even within the same brand [23, 24]. This heterogeneity suggests that the physical characteristics of TiO₂ may differ substantially between, and sometimes within, medicinal products.

Beyond age and gender, the SNDS database lacks information on key cancer risk factors like body mass index or smoking status, as well as on dietary or other sources of exposure to TiO_2_ (e.g., sunscreens, toothpaste). We therefore designed our study to minimize the potential for confounding related to individuals’ characteristics by focusing on populations taking specific medications, assuming that users of the same drug share similarities in diet, cancer risk factors, and other medication use. Once a specific medication (e.g., 1000 mg metformin tablets) is prescribed, the allocation of TiO_2_-containing versus TiO_2_-free formulations is not influenced by the patient’s personal characteristics but rather determined by the pharmacist’s supply, making TiO_2_ exposure essentially random within the same drug, dose, and form.

We carefully addressed potential confounding by other drug constituents. Some excipients might be associated with cancer risk. For instance, a recent epidemiological study linked the ingestion of some food additive emulsifiers (e.g., bee wax and mono- and diglycerides of fatty acids), also used as pharmaceutical excipients, to higher cancer risks [25]. The presence of nanoparticles is also confirmed or suspected in excipients other than TiO_2_, such as calcium carbonate, iron oxides, calcium silicate, synthetic amorphous silicas, microcrystalline cellulose, magnesium stearate, or talc [26]. Data on the potential carcinogenic or anticarcinogenic properties of excipients remains scarce. To address this uncertainty, we selected drugs with formulations allowing us to disentangle the effects of TiO_2_ from other excipients and adjusted analyses for the amounts of other excipients ingested through the selected drug. By matching cases and controls on the date of the first claim for the selected drug, we ensured comparable exposure opportunities to TiO_2_. Furthermore, we adjusted our analyses for the cumulative amount of selected drug’s active ingredients. This is an important precaution since, for example, metformin may be associated with the risk of several cancers [27].

Our study has limitations. First, our data reflects dispensed prescriptions rather than consumed ones. However, the cumulative TiO_2_ consumed dose is likely proportional to the dispensed dose, supporting the validity of our dose-response assessment. Second, cancer identification relied on medico-administrative data. While widely used, this method has limitations: comparisons of cancer incidence rates between registry and SNDS show some discrepancies [28, 29]. The resulting outcome misclassification, likely unrelated to TiO_2_ exposure levels, may have biased our estimates towards the null. Third, information on TiO_2_ presence in pharmaceuticals was limited to the most recent ANSM and Theriaque data (2021). Changes in excipients within formulations are not always reflected in presentation identification codes. Thus, exposure misclassification could have occurred if TiO_2_ content in selected drugs changed between 2006 and 2021. Finally, and most importantly, our study was primarily designed on the assumption that an increase in TiO_2_ ingestion would cause the same variation in cancer risk, whatever the amount of previous exposure. We thus did not take into account sources of TiO_2_ exposure—dietary or pharmaceutical—other than the consumption of the selected drugs, which makes our study not optimal to accurately assess non-linear associations, for example in the presence of a threshold effect. Our results, which indicate no meaningful linear association between increasing TiO_2_ exposure through drug use and cancer risk, yet suggest that non-linear associations may be present for some cancers, i.e., eye/brain/other parts of the central nervous system cancers, pancreatic cancer, and melanoma. The exact shapes of these non-linear associations can not be properly inferred from our study. Our results were obtained among individuals taking certain medications used to treat chronic conditions, who were likely to have been exposed previously to TiO_2_ ingestion through several sources (including dietary and pharmaceutical sources) and thus cannot be extrapolated to individuals without previous TiO_2_ exposure.

## Conclusion

This is the first epidemiological study assessing the association between TiO_2_ ingestion and cancer risk. Our findings revealed no meaningful linear association between increments in TiO_2_ exposure from medications and cancer risk. However, there was some suggestions of non-linear associations for some cancer sites. Further epidemiological research investigating TiO_2_ exposures, ideally derived from various sources (dietary and pharmaceutical), in relation with cancer risk is needed to confirm and refine our findings, especially regarding potential non-linear associations.

## Electronic supplementary material

Below is the link to the electronic supplementary material.

Supplementary Material 1

## Declarations

### Disclaimer

This work represents the own views of the authors and does not necessarily represent the opinion of EPI-PHARE, ANSM nor Cnam. Where authors are identified as personnel of the International Agency for Research on Cancer / World Health Organization, the authors alone are responsible for the views expressed in this article and they do not necessarily represent the decisions, policy or views of the International Agency for Research on Cancer / World Health Organization.
