# Supplementary material for: Ingestion of titanium dioxide as an excipient in medicines and the risk of cancer: a nationwide study within the French National health data system
Source: Eur J Epidemiol. 2025 Jul 2;40(7):833–43. doi: 10.1007/s10654-025-01263-4 (PMC12304061; doi:10.1007/s10654-025-01263-4)
Supplement: Supplementary file 1 — Supplementary Material 1 [file 10654_2025_1263_MOESM1_ESM.pdf]

Ingestion of titanium dioxide as an excipient in medicines and the risk of cancer: a nationwide study within the French National Health Data System

European Journal of Epidemiology

Manon Cairat, Gianluca Severi, Inge Huybrechts, Agnès Fournier

Corresponding author: Agnès Fournier, Université Paris-Saclay, UVSQ, Inserm, Gustave Roussy, CESP, 94805, Villejuif, France; Email: agnes.fournier@gustaveroussy.fr

Supplementary material

Supplementary methods: Selection of drugs

Table S1. Pre-selected {ATC 5th level; dose; form} triplets

Table S2. Main categories and subcategories of cancer localizations and their corresponding ICD-10 codes

Table S3. Geographical region of cancer cases and matched controls, and cancer sites for cases

Table S4. Associations between exposure to TiO_2 and the risk of cancer, overall and by main ICD-10 categories of cancer localizations. Exposure to TiO_2 lagged by 1 year or by 5 years

Table S5. Associations between exposure to TiO_2 (lagged by 5 years) and the risk of cancer, overall and by main ICD-10 categories of cancer localizations, in the two individual case-control studies

Table S6. Associations between exposure to TiO_2 (cumulative number of tablets, lagged by 5 years) and the risk of cancer, overall and by main ICD-10 categories of cancer localizations. Varying adjustment factors

Table S7. Associations between exposure to TiO_2 (lagged by 5 years) and the risk of cancer, overall and by main ICD-10 categories of cancer localizations. Varying the criteria for incident cancer cases identification

Figure S1. Flow-chart for the case-control dataset originating from the metformin cohort

Figure S2. Flow-chart for the case-control dataset originating from the acebutolol 200 mg cohort

Figure S3. Associations between exposure to TiO_2 (lagged by 5 years) and the risk of cancer by subcategories of cancer localizations

Supplementary methods: Selection of drugs

Among 5th-level ATC codes comprising pharmaceutical specialties reimbursed in France over the 2006-2016 period (n = 1389), 114 had at least 10,000 boxes without TiO_2 and 10,000 boxes with TiO_2 reimbursed each year. Of these, 49 comprised {5th-level ATC; dose} pairs with at least 10,000 boxes without TiO_2 and 10,000 boxes with TiO_2 reimbursed each year, and 24 comprised {5th-level ATC; dose; form} triplets with at least 10,000 boxes without TiO_2 and 10,000 boxes with TiO_2 reimbursed each year. Form was categorized as dispersible tablets, effervescent tablets, other tablets, soft capsules, capsules, granules, powder, or other. The corresponding {5th-level ATC; dose; form} triplets are shown in Table S1, which also indicates whether they met exclusion criteria, i.e. either i) they did not have at least 300,000 boxes with TiO_2 and at least 300,000 boxes without TiO_2 reimbursed annually during the 2006-2016 period, or ii) at least one excipient was exclusively present in either TiO_2-containing or TiO_2-free formulations.

Table S1. Pre-selected {5th-level ATC code; dose; form} triplets

	No. TiO_2-containing / TiO_2-free boxes reimbursed in France (thousands) by year*												Exclusion criteria	
**{ATC; dose; form}**	**2006**	**2007**	**2008**	**2009**	**2010**	**2011**	**2012**	**2013**	**2014**	**2015**	**2016**	**2006-2016**	**NOT(≥300,000 TiO_2-containing and ≥300,000 TiO_2-free boxes reimbursed annually)**	**Any excipient exclusively present in either TiO_2-containing or TiO_2-free formulations?**
{N02BE01; 1000 mg; tablet[†]}	14,996 / 30,286	17,634 / 40,931	19,582 / 48,287	23,850 / 61,293	27,367 / 70,337	34,626 / 84,580	50,885 / 124,509	54,252 / 139,394	55,952 / 150,772	53,132 / 157,398	52,559 / 167,151	404,836 / 1,074,939	No	Propylene glycol; glycerol behanate; colloidal anhydrous silica
{M01AE01; 400 mg; tablet[†]}	7182 / 890	8422 / 605	9053 / 567	10,480 / 377	10,059 / 280	11,179 / 283	14,838 / 387	15,766 / 381	15,380 / 367	15,849 / 412	15,943 / 404	134,151 / 4954		No
**{A10BA02; 1000 mg; tablet[†]}**	**3893 / 4950**	**4841 / 4302**	**4726 / 3499**	**4105 / 3290**	**3680 / 3112**	**2671 / 3937**	**5078 / 4006**	**5635 / 3114**	**5379 / 3353**	**5128 / 3461**	**4645 / 3897**	**49,782 / 40,923**	**No**	**No**
**{A10BA02; 850 mg; tablet[†]}**	**3483 / 3984**	**3889 / 3481**	**3501 / 3102**	**2939 / 3005**	**2583 / 2897**	**1637 / 3602**	**2201 / 4703**	**2426 / 4020**	**3894 / 3039**	**3658 / 2951**	**3452 / 2820**	**33,663 / 37,604**	**No**	**No**
{N05BA06; 1 mg; tablet[†]}	361 / 4850	1200 / 3862	2182 / 2678	2184 / 2459	2177 / 2331	1986 / 2331	3092 / 2606	3523 / 1858	3015 / 2145	3220 / 1659	3147 / 1515	26,087 / 28,293	No	Macrogol; talc; polymethyl methacrylate
**{A10BA02; 500 mg; tablet[†]}**	**826 / 1241**	**1154 / 1313**	**1297 / 1389**	**1319 / 1569**	**1358 / 1824**	**953 / 2419**	**1520 / 3648**	**1834 / 3368**	**3845 / 3322**	**3847 / 3242**	**3884 / 3189**	**21,836 / 26,524**	**No**	**No**
{A03AA05; 200 mg; tablet[†]}	2789 / 409	2200 / 1471	1866 / 1848	1998 / 1885	1964 / 1903	1914 / 1671	2518 / 2363	2474 / 2426	2587 / 2467	2754 / 2300	3010 / 2065	26,074 / 20,809	No	Macrogol
{C07AB07; 10 mg; tablet[†]}	3574 / 1078	3374 / 1161	3051 / 1117	2817 / 962	3120 / 500	3252 / 166	4440 / 184	4078 / 190	3795 / 179	3628 / 117	3372 / 132	38,502 / 5787	Yes	Macrogol; hypromellose
**{M01AE01; 200 mg; tablet[†]}**	**2922 / 1723**	**2484 / 2041**	**1996 / 2268**	**2045 / 2278**	**1686 / 1858**	**1673 / 1967**	**1777 / 2613**	**1299 / 2699**	**986 / 2319**	**988 / 2275**	**859 / 2228**	**18,714 / 24,270**	**No**	**No**
{N02BE01; 500 mg; powder}	3236 / 43	3155 / 31	3011 / 24	3502 / 44	2847 / 120	3040 / 191	3870 / 280	4078 / 339	3801 / 236	3916 / 239	4047 / 266	38,503 / 1813	Yes	Sodium benzoate; aspartam
{C07AB03; 50 mg; tablet[†]}	2686 / 910	2651 / 1026	2461 / 1094	2265 / 1006	2129 / 1006	2079 / 931	2858 / 1290	2666 / 1276	2514 / 1290	2391 / 1316	2251 / 1344	26,952 / 12,490	No	Hypromellose
**{C07AB04; 200 mg; tablet[†]}**	**1688 / 1720**	**1989 / 1414**	**1980 / 1328**	**1817 / 1292**	**1697 / 1228**	**1536 / 1196**	**2089 / 1588**	**1995 / 1353**	**1865 / 1229**	**1860 / 1039**	**1745 / 933**	**20,262 / 14,320**	**No**	**No**

{C03EA04; 25mg/15 mg; tablet†}	114 / 3196	94 / 3036	70 / 2824	58 / 2561	46 / 2325	44 / 2093	46 / 2758	31 / 2516	28 / 2319	24 / 2155	21 / 2004	575 / 27,788	Yes	Ammonium methacrylate copolymer; macrogol; hypromellose; corn and rice starch
{G04CA01; 10 mg; tablet†}	89 / 1841	251 / 1721	286 / 1598	304 / 1608	362 / 1617	360 / 1603	550 / 2432	598 / 2395	675 / 2378	879 / 2246	854 / 2347	5208 / 21,785	Yes	Propylene glycol; calcium hydrogen phosphate; hydrogenated vegetable oils
{A03AA05; 100 mg; tablet†}	1142 / 2650	744 / 2521	403 / 2191	413 / 1852	401 / 1583	416 / 1279	439 / 1762	311 / 1786	323 / 1747	273 / 1768	236 / 1783	5100 / 20,921	Yes	Tartric acid
**{G03AA07; 30 µg/150 µg; tablet†}**	**799 / 1069**	**816 / 1091**	**912 / 1043**	**904 / 1051**	**845 / 964**	**760 / 871**	**1030 / 1074**	**1412 / 1256**	**1493 / 1257**	**1490 / 1225**	**1513 / 1196**	**11,975 / 12,097**	**No**	**No**
{C07AB03; 100 mg; tablet†}	1821 / 657	1692 / 677	1477 / 661	1255 / 578	1115 / 549	1040 / 481	1346 / 625	1195 / 580	1079 / 550	975 / 536	870 / 521	13,865 / 6414	No	Hypromellose
{M04AC01; 1 mg; tablet†}	820 / 482	852 / 477	890 / 462	933 / 460	1060 / 492	1119 / 506	1567 / 708	1468 / 702	1456 / 725	1422 / 755	1359 / 786	12,945 / 6554	No	Erythrosine; patent blue; propylene glycol; hypromellose; glucose; corn starch; saccharose
{A03AX04; 100 mg; tablet†}	586 / 1599	1119 / 843	1205 / 442	1059 / 455	932 / 427	820 / 381	1065 / 439	1064 / 333	1016 / 307	1002 / 264	1002 / 175	10,871 / 5664	Yes	Butyl methacrylate copolymer; macrogol; hypromellose; sodium laurilsulfate; stearic acid; lactose; polyvinyl alcohol
{C10AB05; 160 mg; tablet†}	2539 / 327	1223 / 773	828 / 776	694 / 628	617 / 569	548 / 514	750 / 629	641 / 544	561 / 507	524 / 480	390 / 565	9317 / 6312	No	Colloidal anhydrous silica
**{J01AA02; 100 mg; tablet†}**	**758 / 536**	**761 / 547**	**673 / 626**	**628 / 638**	**596 / 617**	**550 / 627**	**745 / 908**	**937 / 710**	**1047 / 474**	**862 / 678**	**810 / 778**	**8368 / 7139**	**No**	**No**
{N03AF01; 200 mg; tablet†}	762 / 439	680 / 476	650 / 467	628 / 443	676 / 386	707 / 327	950 / 377	890 / 416	892 / 402	896 / 415	870 / 411	8599 / 4559	No	Iron oxide yellow; iron oxide red; hypromellose
{N03AF01; 400 mg; tablet†}	945 / 186	876 / 239	857 / 241	825 / 243	915 / 140	937 / 92	1126 / 117	1089 / 133	1075 / 123	1011 / 152	1024 / 133	10,681 / 1799	Yes	Iron oxide yellow; iron oxide red; ethylcellulose; hypromellose
{C08DB01; 300 mg; capsule}	1220 / 18	1180 / 17	1095 / 15	991 / 13	920 / 12	834 / 16	1113 / 31	1012 / 27	931 / 13	788 / 68	667 / 102	10,752 / 332	Yes	No
{C07AB02; 100 mg; tablet†}	170 / 521	190 / 512	206 / 472	183 / 454	166 / 456	146 / 458	180 / 676	136 / 688	124 / 675	110 / 663	98 / 641	1708 / 6215	Yes	Macrogol; glycerol hydroxystearate; talc
{A12AX ; 600 mg/400UI ; tablet†}	320 / 30	393 / 27	419 / 23	451 / 22	494 / 18	517 / 16	729 / 16	722 / 12	696 / 11	708 / 11	710 / 10	6158 / 195	Yes	Povidone; crospovidone; iron oxide yellow, black, red; all-rac-alpha-tocopherol; butylated hydroxytoluene; mannitol; microcrystalline

														cellulose; hyprolose; sodium carboxymethyl starch; medium-chain triglycerides; sodium laurilsulfate; sodium silicate; talc; aluminium silicate; paraffin; xylitol; partially hydrogenated soybean oil
{L02BA01; 20 mg; tablet†}	235 / 299	178 / 292	148 / 295	130 / 296	122 / 304	130 / 301	190 / 400	222 / 380	246 / 364	213 / 408	210 / 411	2025 / 3749	Yes	Macrogol; hypromellose
{C07AB04; 400 mg; tablet†}	307 / 327	341 / 255	328 / 227	291 / 215	262 / 190	233 / 173	307 / 224	286 / 187	263 / 164	246 / 141	231 / 118	3097 / 2219	Yes	No
{C08DA01; 120 mg; tablet†}	111 / 184	109 / 235	125 / 269	132 / 233	139 / 206	133 / 190	210 / 285	231 / 318	234 / 311	250 / 319	256 / 347	1930 / 2899	Yes	Povidone; macrogol; glycerol; lactose
{C08DA01; 40 mg; tablet†}	192 / 26	180 / 41	155 / 60	156 / 588	156 / 58	139 / 69	178 / 109	151 / 131	241 / 27	221 / 52	191 / 81	1958 / 713	Yes	Povidone; glycerol; lactose
{A07EC02; 500 mg; tablet†}	59 / 226	55 / 217	53 / 205	51 / 193	49 / 187	47 / 175	63 / 211	60 / 182	57 / 160	55 / 143	52 / 126	602 / 2027	Yes	Methacrylic acid and ethyl acrylate copolymer; methacrylic acid and methyl methacrylate copolymer; macrogol; iron oxide yellow and red; ethylcellulose; sodium carboxymethyl starch; calcium stearate; magnesium stearate; sodium carbonate; colloidal anhydrous silica; glycine; dibutyl sebacate
{R03DA04; 300 mg; capsule}	312 / 30	273 / 25	226 / 21	186 / 22	171 / 19	150 / 17	177 / 21	156 / 18	138 / 16	122 / 15	107 / 13	2018 / 216	Yes	No
{R03DA04; 200 mg; capsule}	248 / 77	219 / 66	183 / 55	152 / 52	140 / 47	124 / 41	154 / 52	136 / 45	121 / 40	107 / 36	94 / 33	1677 / 544	Yes	Carmin indigo
{N03AX12; 600 mg; tablet†}	47 / 163	84 / 99	112 / 54	104 / 51	103 / 50	100 / 52	134 / 62	144 / 54	149 / 54	161 / 44	151 / 60	1288 / 742	Yes	Macrogol
{N03AX12; 800 mg; tablet†}	22 / 132	52 / 81	80 / 45	76 / 43	74 / 43	69 / 43	90 / 51	95 / 45	99 / 37	104 / 35	94 / 45	857 / 602	Yes	Macrogol; poloxamere
{L02BA01; 10 mg; tablet†}	26 / 21	18 / 20	12 / 20	11 / 19	11 / 17	11 / 17	16 / 23	21 / 21	24 / 27	26 / 28	28 / 33	204 / 248	Yes	Macrogol; hypromellose

* From: Assurance Maladie. Bases de données (Open Data). 2024. http://open-data-assurance-maladie.ameli.fr/medicaments/index.php. [Accessed 31 December 2024].

† neither dispersible nor effervescent

Table S2. Main categories and subcategories of cancer localizations and their corresponding ICD-10 codes

Cancer localizations	ICD-10 Codes
All cancers	C, D0
Lip, oral cavity, and pharynx	C00-C14, D00, D000
Digestive organs	C15-C26, D01, D001, D002
- Colon	- C18, D010
- Liver	- C22
- Pancreas	- C25
Respiratory and intrathoracic organs	C30-C39, D02
- Bronchus and lung	- C34, D022
Bone and articular cartilage	C40-C41
Skin	C43-C44, D03, D04
- Melanoma	- C43, D03
Mesothelial and soft tissue	C45-C49
Breast	C50, D05
Female genital organs	C50-C58, D05, D06, D07, D070, D071, D072, D073
- Corpus uteri	- C54, D070
- Ovary	- C56
Male genital organs	C60-C63, D074, D075, D076
- Prostate	- C61, D075
Urinary tract	C64-C68, D090, D091
- Kidney	- C64
- Bladder	- C67, D090
Eye, brain, and other parts of the central nervous system	C69-C72, D092
- Brain	- C71
Thyroid and other endocrine glands	C73-C75, D093
- Thyroid	- C73, D093
Primary lymphoid, hematopoietic and related tissue	C81-C96
- Multiple myeloma	- C90
- Lymphoid leukemia	- C91
Malignant tumors of ill-defined, secondary, and unspecified sites, and malignant neoplasms of independent (primary) multiple sites	C76-C80, C97, D09, D097, D099

ICD, International Classification of Diseases

Table S3. Geographical region of cancer cases and matched controls, and cancer sites for cases

	Case-control study with exposure measured as cumulative number of tablets containing TiO_2, lagged by 5 years		Case-control study with exposure measured as cumulative dose of TiO_2 in milligrams, lagged by 5 years	
	**Cases n = 293,101**	**Controls n = 2,930,633**	**Cases n = 218,611**	**Controls n = 2,185,643**
**Region**				
Auvergne-Rhône Alpes	32,009 (10.9%)	315,520 (10.8%)	23,987 (11.0%)	235,553 (10.8%)
Bretagne	10,485 (3.6%)	91,540 (3.1%)	7351 (3.4%)	63,698 (2.9%)
Bourgogne-Franche Comté	15,527 (5.3%)	149,680 (5.1%)	11,628 (5.3%)	111,975 (5.1%)
Centre-Val de Loire	12,849 (4.4%)	128,360 (4.4%)	9259 (4.2%)	92,941 (4.3%)
Corse	946 (0.3%)	9337 (0.3%)	750 (0.3%)	7463 (0.3%)
Grand Est	25,974 (8.9%)	253,337 (8.7%)	20,502 (9.4%)	199,700 (9.2%)
Guadeloupe	2228 (0.8%)	29,384 (1.0%)	1754 (0.8%)	23,450 (1.1%)
Guyane	393 (0.1%)	6561 (0.2%)	297 (0.1%)	4757 (0.2%)
Hauts de France	29,186 (10.0%)	274,198 (9.4%)	23,155 (10.6%)	218,157 (10.0%)
Ile de France	58,816 (20.1%)	651,498 (22.3%)	40,963 (18.8%)	455,730 (20.9%)
La Réunion	3282 (1.1%)	48,022 (1.6%)	2428 (1.1%)	36,400 (1.7%)
Martinique	1773 (0.6%)	22,940 (0.8%)	1368 (0.6%)	18,046 (0.8%)
Mayotte	95 (0.03%)	2644 (0.1%)	94 (0.04%)	2512 (0.1%)
Normandie	16,019 (5.5%)	145,681 (5.0%)	12,319 (5.6%)	111,526 (5.1%)
Nouvelle Aquitaine	22,334 (7.6%)	210,780 (7.2%)	17,531 (8.0%)	165,409 (7.6%)
Occitanie	20,770 (7.1%)	207,631 (7.1%)	16,048 (7.4%)	160,376 (7.3%)
Pays de Loire	15,453 (5.3%)	137,544 (4.7%)	10,487 (4.8%)	92,639 (4.2%)
Provence Alpes Côte d'Azur	24,610 (8.4%)	242,134 (8.3%)	18,392 (8.4%)	181,952 (8.3%)
Missing	352	3842	298	3359
**Cancer site**				
Lip, oral cavity and pharynx	6150 (2.1%)	-	4502 (2.1%)	-
Digestive organs	72,654 (24.8%)	-	54,741 (25.0%)	-
Respiratory and intrathoracic organs	28,064 (9.6%)	-	21,017 (9.6%)	-
Bone and articular cartilage	580 (0.2%)	-	424 (0.2%)	-
Melanoma and other malignant neoplasms of skin	42,467 (14.5%)	-	30,891 (14.1%)	-
Mesothelial and soft tissue	2763 (0.9%)	-	2052 (0.9%)	-
Breast	25,620 (8.7%)	-	19,090 (8.7%)	-
Female genital organs	10,993 (3.7%)	-	8284 (3.8%)	-
Male genital organs	34,195 (11.7%)	-	25,404 (11.6%)	-
Urinary tract	28,032 (9.6%)	-	21,069 (9.6%)	-
Eye, brain and other parts of central nervous system	4076 (1.4%)	-	3077 (1.4%)	-
Thyroid and other endocrine glands	3852 (1.3%)	-	2,963 (1.4%)	-
Ill-defined, secondary and unspecified sites	12,438 (4.2%)	-	9,344 (4.3%)	-
Lymphoid, hematopoietic and related tissue	21,217 (7.2%)	-	15,753 (7.2%)	-

Table S4. Associations between exposure to TiO_2 and the risk of cancer, overall and by main ICD-10 categories of cancer localizations. Exposure to TiO_2 lagged by 1 year or by 5 years

	5-year lag		1-year lag		5-year lag		1-year lag	
	**No. cases**	**RR* (95% CI) per increment of 1000 tablets containing TiO_2**	**No. cases**	**RR* (95% CI) per increment of 1000 tablets containing TiO_2**	**No. cases**	**RR* (95% CI) per increment of 10,000 mg of TiO_2**	**No. cases**	**RR* (95% CI) per increment of 10,000 mg of TiO_2**
All cancers	293,101	1.00 (0.99-1.01)	397,399	1.00 (1.00-1.00)	218,611	1.00 (0.99-1.01)	292,480	1.00 (0.99-1.00)
Lip, oral cavity and pharynx	6150	0.99 (0.94-1.04)	8799	1.01 (0.98-1.05)	4502	0.99 (0.93-1.06)	6468	1.02 (0.98-1.07)
Digestive organs	72,654	1.00 (0.99-1.01)	97,461	1.00 (0.99-1.01)	54,741	1.00 (0.98-1.01)	72,459	1.00 (0.98-1.01)
Respiratory and intrathoracic organs	28,064	1.00 (0.98-1.02)	38,676	1.01 (0.99-1.02)	21,017	1.01 (0.98-1.03)	28,692	1.00 (0.98-1.02)
Bone and articular cartilage	580	0.93 (0.81-1.06)	819	0.95 (0.86-1.05)	424	0.91 (0.75-1.09)	602	0.95 (0.84-1.08)
Skin	42,467	1.01 (0.99-1.02)	55,317	1.01 (1.00-1.02)	30,891	1.01 (0.99-1.04)	39,583	1.01 (0.99-1.02)
Mesothelial and soft tissue	2763	1.04 (0.97-1.11)	3741	1.02 (0.97-1.07)	2052	1.03 (0.95-1.12)	2730	1.00 (0.94-1.07)
Breast	25,057	1.01 (0.99-1.04)	35,454	1.01 (0.99-1.02)	18,668	1.03 (1.00-1.07)	26,109	1.01 (0.99-1.03)
Female genital organs	10,990	1.02 (0.98-1.06)	15,418	1.02 (0.99-1.04)	8282	1.02 (0.98-1.07)	11,417	1.01 (0.98-1.04)
Male genital organs	34,190	0.99 (0.97-1.01)	46,990	0.99 (0.98-1.00)	25,402	0.98 (0.96-1.01)	34,435	1.00 (0.98-1.01)
Urinary tract	28,032	1.01 (0.99-1.03)	37,709	1.00 (0.99-1.01)	21,069	1.00 (0.98-1.03)	27,948	1.00 (0.98-1.01)
Eye, brain and other parts of the CNS	4076	1.02 (0.96-1.08)	5691	1.02 (0.98-1.06)	3077	0.99 (0.92-1.07)	4250	1.01 (0.96-1.06)
Thyroid and other endocrine glands	3852	0.96 (0.91-1.02)	5781	0.95 (0.91-0.99)	2963	0.97 (0.90-1.05)	4396	0.95 (0.90-1.00)
Lymphoid, hematopoietic and related tissue	21,217	0.97 (0.95-1.00)	28,470	0.98 (0.96-0.99)	15,753	0.99 (0.96-1.02)	20,791	0.98 (0.96-1.00)

CI, confidence interval; CNS, central nervous system; RR, relative risk.
*Adjusted for age, sex, cohort of origin (metformin / acebutolol) and time since first use of the selected drug (metformin or acebutolol, depending on the cohort of origin) (matching factors), and region, social deprivation index, cumulative numbers of tablets containing the constituents listed in Table 1.

Table S5. Associations between exposure to TiO_2 (lagged by 5 years) and the risk of cancer, overall and by main ICD-10 categories of cancer localizations, in the two individual case-control studies

	Case-control study nested in the cohort of metformin users				Case-control study nested in the cohort of 200 mg acebutolol users			
	**No. cases**	**RR* (95% CI) per increment of 1000 tablets containing TiO_2**	**No. cases**	**RR* (95% CI) per increment of 10,000 mg of TiO_2**	**No. cases**	**RR* (95% CI) per increment of 1000 tablets containing TiO_2**	**No. cases**	**RR* (95% CI) per increment of 10,000 mg of TiO_2**
All cancers	231,167	1.00 (0.99-1.00)	180,345	1.00 (0.99-1.01)	61,934	1.01 (0.99-1.03)	38,266	1.25 (0.88-1.76)
Lip, oral cavity and pharynx	4685	1.00 (0.95-1.05)	3630	0.99 (0.93-1.06)	1465	0.94 (0.81-1.08)	872	0.61 (0.07-5.67)
Digestive organs	60,418	1.00 (0.99-1.01)	47,236	1.00 (0.98-1.01)	12,236	1.02 (0.97-1.07)	7505	1.80 (0.83-3.91)
Respiratory and intrathoracic organs	22,109	1.00 (0.97-1.02)	17,285	1.00 (0.98-1.03)	5955	1.01 (0.94-1.08)	3732	1.95 (0.66-5.77)
Bone and articular cartilage	455	0.93 (0.80-1.08)	356	0.90 (0.75-1.09)	125	0.91 (0.59-1.41)	68	NOT ESTIMABLE
Skin	30,565	1.00 (0.98-1.02)	23,607	1.01 (0.99-1.04)	11,902	1.04 (0.99-1.09)	7284	1.82 (0.83-3.96)
Mesothelial and soft tissue	2157	1.03 (0.96-1.10)	1656	1.03 (0.94-1.12)	606	1.05 (0.80-1.38)	396	NOT ESTIMABLE
Breast	19,591	1.01 (0.99-1.04)	15,200	1.03 (1.00-1.07)	5466	1.00 (0.93-1.08)	3468	0.85 (0.25-2.92)
Female genital organs	8943	1.02 (0.98-1.05)	6994	1.02 (0.97-1.07)	2047	1.07 (0.93-1.23)	1288	3.33 (0.33-33.5)
Male genital organs	26,663	0.99 (0.97-1.00)	20,795	0.98 (0.96-1.01)	7527	0.99 (0.93-1.06)	4607	0.75 (0.28-2.05)
Urinary tract	22,298	1.01 (0.99-1.03)	17,551	1.00 (0.98-1.03)	5734	0.96 (0.90-1.03)	3518	0.41 (0.13-1.29)
Eye, brain and other parts of the CNS	3234	1.02 (0.96-1.08)	2523	0.99 (0.92-1.07)	842	1.12 (0.92-1.36)	554	0.65 (0.02-18.1)
Thyroid and other endocrine glands	3185	0.95 (0.90-1.02)	2531	0.97 (0.90-1.05)	667	1.03 (0.78-1.34)	432	NOT ESTIMABLE
Lymphoid, hematopoietic and related tissue	16,431	0.97 (0.95-1.00)	12,801	0.99 (0.96-1.01)	4786	1.00 (0.93-1.08)	2952	1.04 (0.31-3.46)

CI, confidence interval; CNS, central nervous system; RR, relative risk.

*Adjusted for age, sex, time since first use of the selected drug (metformin or acebutolol, depending on the cohort of origin) (matching factors), and region, social deprivation index, cumulative numbers of tablets containing the constituents listed in Table 1.

Table S6. Associations between exposure to TiO_2 (cumulative number of tablets, lagged by 5 years) and the risk of cancer, overall and by main ICD-10 categories of cancer localizations. Varying adjustment factors

	RR* (95% CI) per increment of 1000 tablets containing TiO_2			
	**Model 1**[1]	**Model 2**[2]	**Model 3**[3]	**Model 4**[4]
All cancers	1.00 (0.99-1.01)	1.00 (0.99-1.01)	1.00 (1.00-1.01)	1.01 (1.01-1.02)
Lip, oral cavity and pharynx	0.99 (0.94-1.04)	0.99 (0.94-1.04)	1.02 (0.99-1.05)	1.02 (1.00-1.04)
Digestive organs	1.00 (0.99-1.01)	1.00 (0.99-1.01)	1.00 (1.00-1.01)	1.03 (1.02-1.03)
Respiratory and intrathoracic organs	1.00 (0.98-1.02)	1.00 (0.98-1.02)	1.01 (0.99-1.02)	1.02 (1.01-1.03)
Bone and articular cartilage	0.93 (0.81-1.06)	0.92 (0.80-1.06)	1.00 (0.92-1.08)	1.02 (0.95-1.08)
Skin	1.01 (0.99-1.02)	1.01 (0.99-1.02)	1.01 (1.00-1.02)	1.02 (1.01-1.03)
Mesothelial and soft tissue	1.04 (0.97-1.11)	1.04 (0.98-1.11)	1.03 (0.99-1.07)	1.02 (0.99-1.05)
Breast	1.01 (0.99-1.04)	1.01 (0.99-1.04)	1.01 (0.99-1.02)	0.99 (0.98-1.00)
Female genital organs	1.02 (0.98-1.06)	1.02 (0.98-1.06)	1.02 (1.00-1.04)	1.01 (0.99-1.03)
Male genital organs	0.99 (0.97-1.01)	0.98 (0.97-1.00)	0.99 (0.98-1.00)	0.99 (0.99-1.00)
Urinary tract	1.01 (0.99-1.03)	1.01 (0.99-1.03)	1.00 (0.99-1.01)	1.01 (1.00-1.02)
Eye, brain and other parts of the CNS	1.02 (0.96-1.08)	1.02 (0.97-1.08)	0.99 (0.96-1.02)	1.00 (0.98-1.03)
Thyroid and other endocrine glands	0.96 (0.91-1.02)	0.96 (0.90-1.02)	0.98 (0.95-1.02)	1.00 (0.97-1.03)
Lymphoid, hematopoietic and related tissue	0.97 (0.95-1.00)	0.97 (0.95-1.00)	0.99 (0.98-1.01)	1.01 (0.99-1.02)

CI, confidence interval; CNS, central nervous system; RR, relative risk.

[1] Adjusted for age, sex, cohort of origin (metformin / acebutolol) and time since first use of the selected drug (metformin or acebutolol, depending on the cohort of origin) (matching factors), and region, social deprivation index, cumulative numbers of tablets containing the constituents listed in Table 1.

[2] Adjusted for age, sex, cohort of origin (metformin / acebutolol) and time since first use of the selected drug (metformin or acebutolol, depending on the cohort of origin) (matching factors), and cumulative numbers of tablets containing the constituents (excipients and active ingredients) listed in Table 1.

[3] Adjusted for age, sex, cohort of origin (metformin / acebutolol) and time since first use of the selected drug (metformin or acebutolol, depending on the cohort of origin) (matching factors), and cumulative numbers of tablets containing the active ingredients listed in Table 1.

[4] Adjusted for age, sex, cohort of origin (metformin / acebutolol) and time since first use of the selected drug (metformin or acebutolol, depending on the cohort of origin) (matching factors).

Table S7. Associations between exposure to TiO_2 (lagged by 5 years) and the risk of cancer, overall and by main ICD-10 categories of cancer localizations. Varying the criteria for incident cancer cases identification

**Data used to identifiy incident cancer cases**	**"Main", "related" and "associated" diagnoses at hospital discharges and LTD registrations**		**"Main" and "related" diagnoses at hospital discharges and LTD registrations**		**"Main", "related" and "associated" diagnoses at hospital discharges and LTD registrations**		**"Main" and "related" diagnoses at hospital discharges and LTD registrations**	
	**No. cases**	**RR* (95% CI) per increment of 1000 tablets containing TiO_2**	**No. cases**	**RR* (95% CI) per increment of 1000 tablets containing TiO_2**	**No. cases**	**RR* (95% CI) per increment of 10,000 mg of TiO_2**	**No. cases**	**RR* (95% CI) per increment of 10,000 mg of TiO_2**
All cancers	293,101	1.00 (0.99-1.01)	273,958	1.00 (0.99-1.01)	218,611	1.00 (0.99-1.01)	204,286	1.00 (0.99-1.00)
Lip, oral cavity and pharynx	6150	0.99 (0.94-1.04)	6012	1.01 (0.96-1.06)	4502	0.99 (0.93-1.06)	4395	0.99 (0.93-1.05)
Digestive organs	72,654	1.00 (0.99-1.01)	68,741	1.01 (0.99-1.02)	54,741	1.00 (0.98-1.01)	51,841	0.99 (0.98-1.01)
Respiratory and intrathoracic organs	28,064	1.00 (0.98-1.02)	27,037	1.00 (0.98-1.02)	21,017	1.01 (0.98-1.03)	20,218	1.00 (0.98-1.03)
Bone and articular cartilage	580	0.93 (0.81-1.06)	519	1.00 (0.87-1.16)	424	0.91 (0.75-1.09)	375	1.04 (0.87-1.25)
Skin	42,467	1.01 (0.99-1.02)	40,679	1.01 (0.99-1.03)	30,891	1.01 (0.99-1.04)	26,606	1.00 (0.98-1.02)
Mesothelial and soft tissue	2763	1.04 (0.97-1.11)	2569	1.03 (0.96-1.10)	2052	1.03 (0.95-1.12)	1918	1.01 (0.93-1.11)
Breast	25,057	1.01 (0.99-1.04)	24,082	1.02 (0.99-1.04)	18,668	1.03 (1.00-1.07)	17,936	1.02 (0.99-1.06)
Female genital organs	10,990	1.02 (0.98-1.06)	10,505	1.01 (0.98-1.05)	8282	1.02 (0.98-1.07)	7909	1.03 (0.98-1.08)
Male genital organs	34,190	0.99 (0.97-1.01)	32,336	0.98 (0.96-0.99)	25,402	0.98 (0.96-1.01)	23,994	0.98 (0.96-1.00)
Urinary tract	28,032	1.01 (0.99-1.03)	26,732	1.01 (0.99-1.03)	21,069	1.00 (0.98-1.03)	20,073	1.01 (0.98-1.04)
Eye, brain and other parts of the CNS	4076	1.02 (0.96-1.08)	3706	1.04 (0.98-1.10)	3077	0.99 (0.92-1.07)	2797	1.00 (0.93-1.08)
Thyroid and other endocrine glands	3852	0.96 (0.91-1.02)	3578	0.99 (0.93-1.05)	2963	0.97 (0.90-1.05)	2751	0.97 (0.89-1.04)
Lymphoid, hematopoietic and related tissue	21,217	0.97 (0.95-1.00)	17,704	0.97 (0.95-1.00)	15,753	0.99 (0.96-1.02)	13,163	0.97 (0.94-1.00)

CI, confidence interval; CNS, central nervous system; LTD, long-term disease; RR, relative risk.

*Adjusted for age, sex, cohort of origin (metformin / acebutolol) and time since first use of the selected drug (metformin or acebutolol, depending on the cohort of origin) (matching factors), and region, social deprivation index, cumulative numbers of tablets containing the constituents listed in table 1.

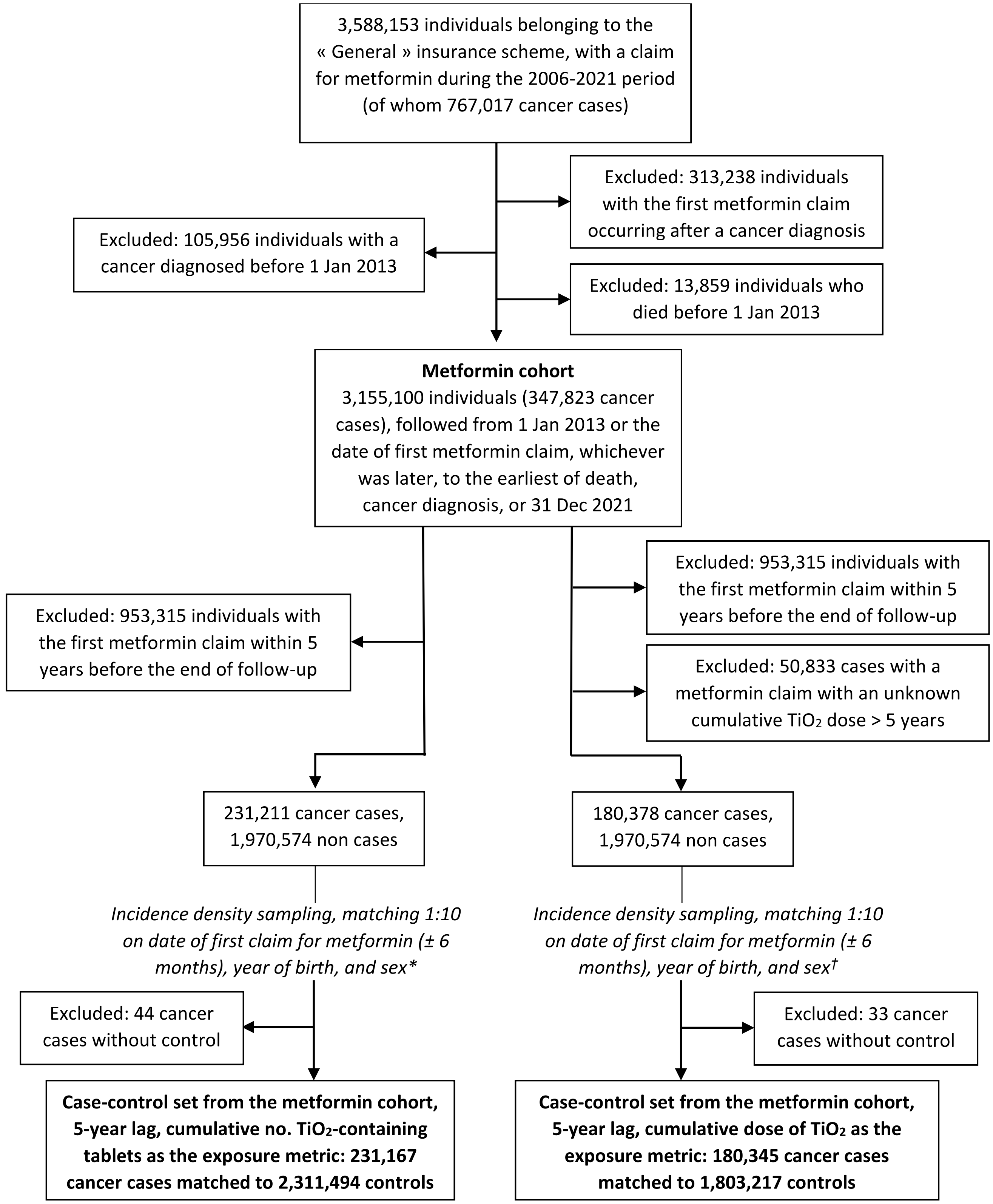

Figure S1. Flow-chart for the case-control dataset originating from the metformin cohort

* Controls were required to be cancer-free on the case's diagnosis date. Cases could serve as controls prior to their diagnosis. An individual could serve as control for several cases.

† Controls were required to be cancer-free and to have a known cumulative TiO_2 dose on the case's diagnosis date. Cases could serve as controls prior to their diagnosis. An individual could serve as control for several cases.

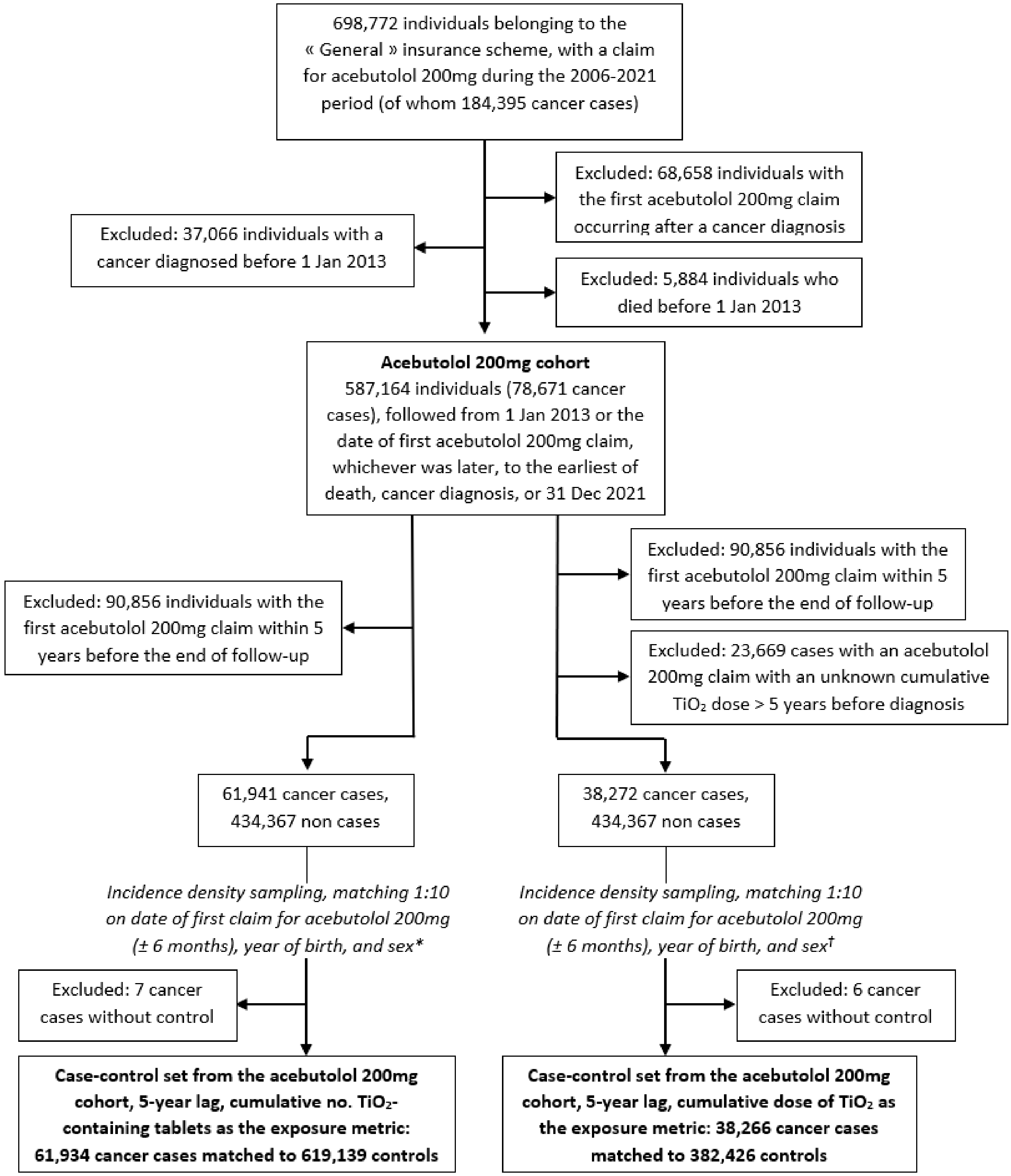

Figure S2. Flow-chart for the case-control dataset originating from the acebutolol 200 mg cohort

* Controls were required to be cancer-free on the case's diagnosis date. Cases could serve as controls prior to their diagnosis. An individual could serve as control for several cases.

† Controls were required to be cancer-free and to have a known cumulative TiO_2 dose on the case's diagnosis date. Cases could serve as controls prior to their diagnosis. An individual could serve as control for several cases.

Exposure measured as cumulative number of tablets containing TiO_2, lagged by 5 years

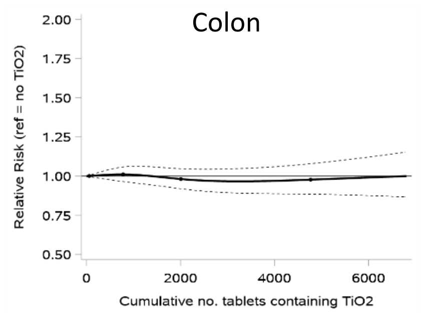

RR* per 1000 tablets: 1.00 (0.98-1.02)
No. cases†: 5749 / 10,439 / 5950 / 2020 / 1039

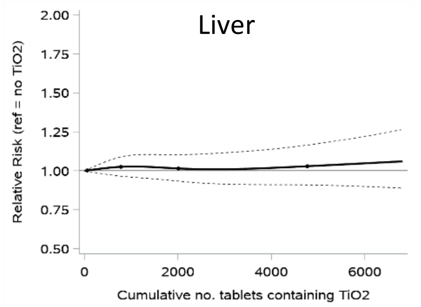

RR* per 1000 tablets: 1.01 (0.98-1.03)
No. cases†: 3109 / 5634 / 3508 / 1341 / 689

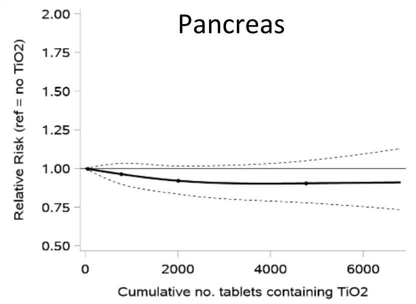

RR* per 1000 tablets: 0.98 (0.95-1.01)
No. cases†: 2556 / 4635 / 2507 / 936 / 467

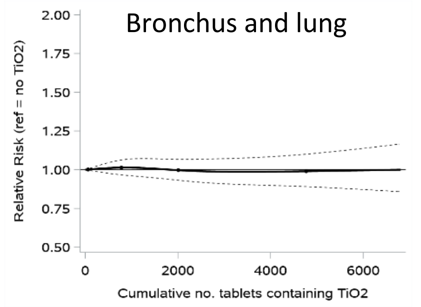

RR* per 1000 tablets: 1.00 (0.98-1.02)
No. cases†: 5514 / 10,466 / 5791 / 1883 / 882

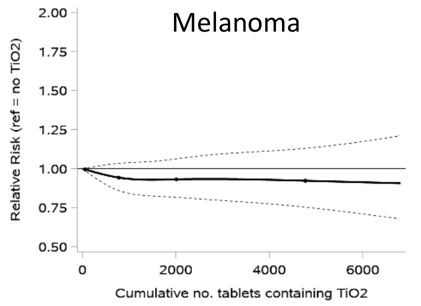

RR* per 1000 tablets: 0.99 (0.95-1.03)
No. cases†: 1541 / 2811 / 1435 / 563 / 261

Exposure measured as cumulative dose of TiO_2 in milligrams, lagged by 5 years

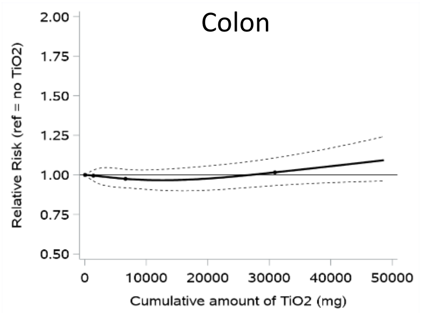

RR* per 10,000 mg: 1.02 (0.99-1.04)
No. cases†: 5987 / 10,223 / 1295 / 977 / 460

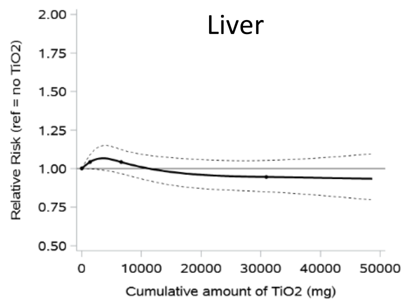

RR* per 10,000 mg: 0.98 (0.95-1.01)
No. cases†: 3225 / 5771 / 822 / 707 / 284

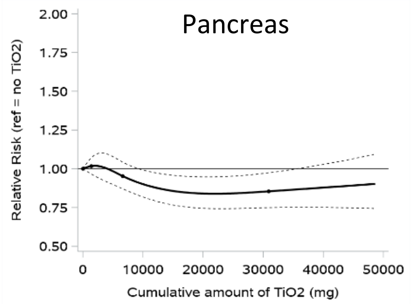

RR* per 10,000 mg: 0.97 (0.93-1.01)
No. cases†: 2659 / 4552 / 512 / 432 / 212

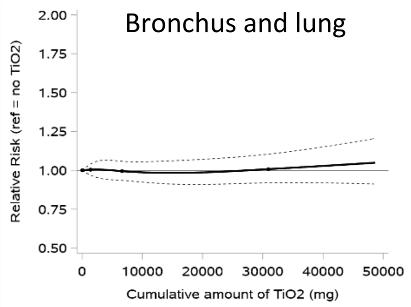

RR* per 10,000 mg: 1.01 (0.98-1.03)
No. cases†: 5721 / 10,255 / 1169 / 903 / 344

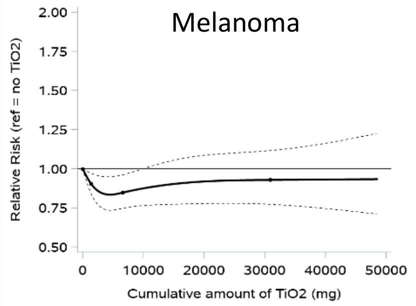

RR* per 10,000 mg: 1.00 (0.95-1.06)
No. cases†: 1614 / 2614 / 282 / 219 / 104

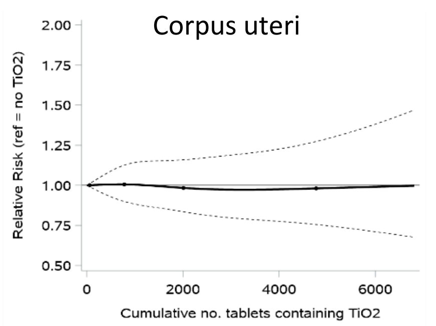

RR* per 1000 tablets: 1.00 (0.94-1.05)
No. cases†: 1215 / 2055 / 1006 / 305 / 133

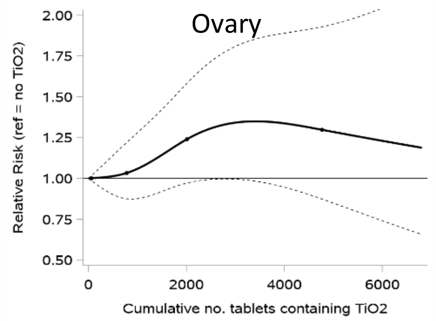

RR* per 1000 tablets: 1.05 (0.97-1.14)
No. cases†: 568 / 999 / 446 / 162 / 53

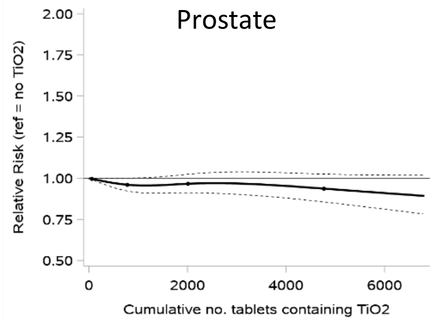

RR* per 1000 tablets: 0.99 (0.97-1.01)
No. cases†: 7494 / 14,304 / 7981 / 2556 / 1128

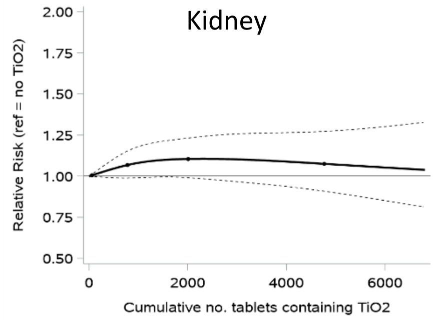

RR* per 1000 tablets: 1.01 (0.97-1.04)
No. cases†: 2017 / 3742 / 2197 / 762 / 335

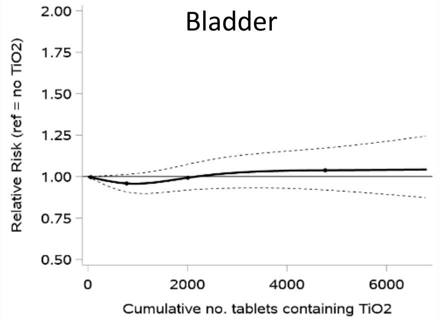

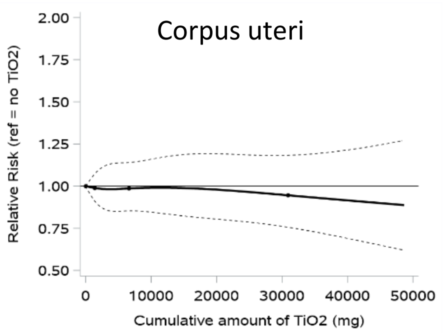

RR* per 10,000 mg: 0.98 (0.92-1.05)
No. cases†: 1268 / 1884 / 210 / 152 / 57

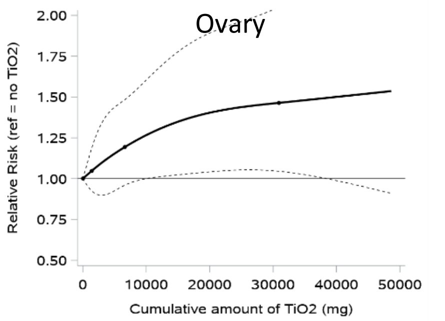

RR* per 10,000 mg: 1.10 (1.00-1.22)
No. cases†: 587 / 868 / 90 / 76 / 21

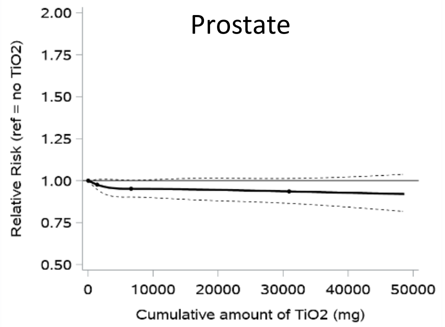

RR* per 10,000 mg: 0.99 (0.96-1.01)
No. cases†: 7807 / 13,690 / 1585 / 1297 / 460

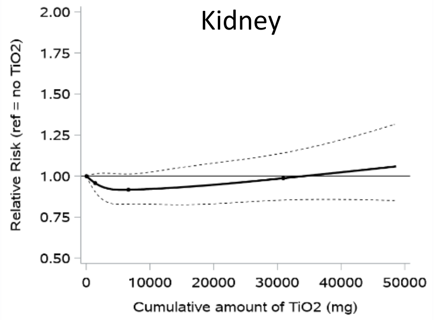

RR* per 10,000 mg: 1.01 (0.97-1.06)
No. cases†: 2099 / 3679 / 491 / 406 / 158

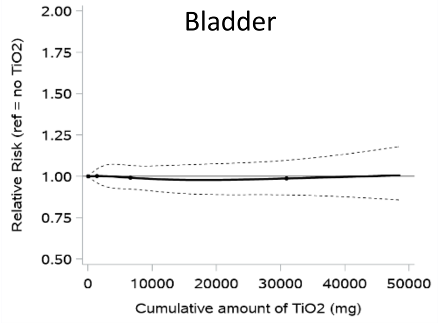

RR* per 1000 tablets: 1.01 (0.98-1.03)
No. cases[†]: 4113 / 7411 / 4285 / 1410 / 627

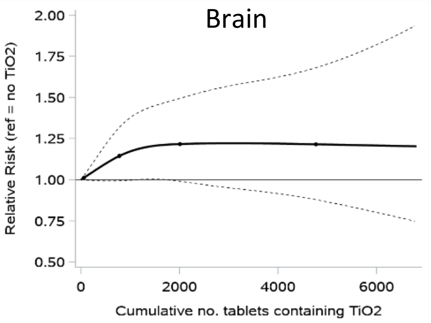

RR* per 1000 tablets: 1.03 (0.97-1.11)
No. cases[†]: 663 / 1203 / 692 / 180 / 86

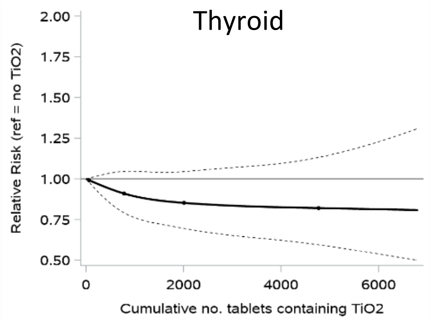

RR* per 1000 tablets: 0.96 (0.90-1.03)
No. cases[†]: 829 / 1519 / 616 / 176 / 84

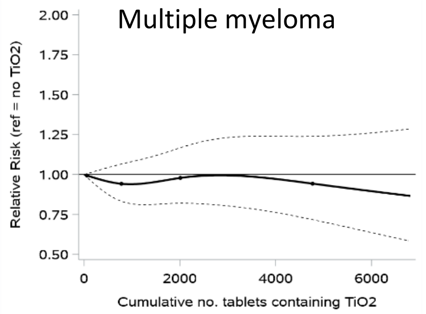

RR* per 1000 tablets: 0.99 (0.93-1.04)
No. cases[†]: 878 / 1654 / 869 / 295 / 131

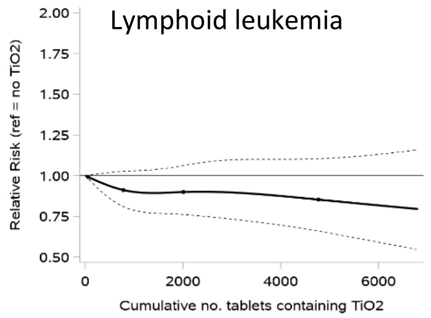

RR* per 1000 tablets: 0.97 (0.92-1.02)
No. cases[†]: 990 / 1640 / 863 / 280 / 131

RR* per 10,000 mg: 1.00 (0.97-1.03)
No. cases[†]: 4272 / 7326 / 851 / 692 / 258

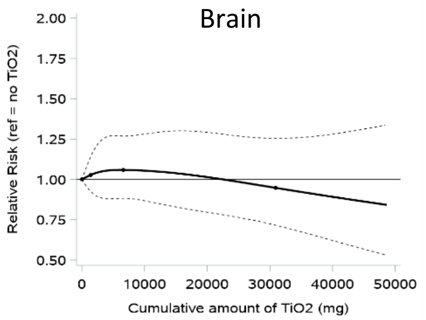

RR* per 10,000 mg: 0.97 (0.89-1.06)
No. cases[†]: 693 / 1175 / 139 / 101 / 38

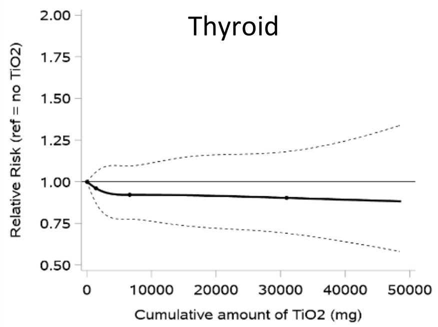

RR* per 10,000 mg: 0.98 (0.90-1.06)
No. cases[†]: 867 / 1349 / 140 / 104 / 32

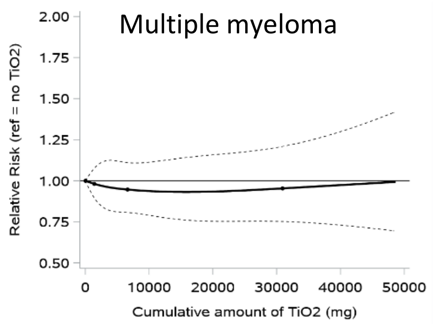

RR* per 10,000 mg: 1.00 (0.93-1.07)
No. cases[†]: 912 / 1539 / 155 / 145 / 60

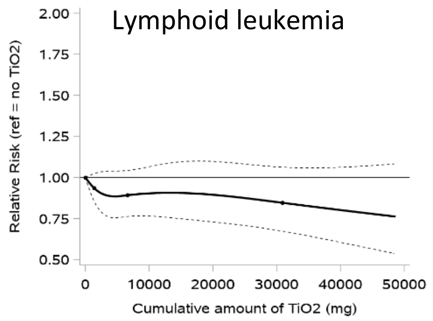

RR* per 10,000 mg: 0.95 (0.89-1.02)
No. cases[†]: 1022 / 1514 / 161 / 155 / 55

Figure S3. Associations between exposure to TiO_2 (lagged by 5 years) and the risk of cancer by subcategories of cancer localizations

* Adjusted for age, sex, cohort of origin (metformin / acebutolol) and time since first use of the selected drug (metformin or acebutolol, depending on the cohort of origin) (matching factors), and region, social deprivation index, cumulative numbers of tablets containing the constituents listed in Table 1.

[†] Number of cases among individuals exposed to 0 / 1-1000 / 1001-3000 / 3001-5000 / 5001+ tablets containing TiO_2 (left column) or to 0 / 1-10,000 / 10,001-20,000 / 20,001-40,000 / 40,001+ mg of TiO2 (right column)
